# Analysis of polarization features of human breast cancer tissue by Mueller matrix visualization

**DOI:** 10.1117/1.JBO.29.5.052917

**Published:** 2024-01-13

**Authors:** Thi-Thu-Hien Pham, Thao-Ngan Ngoc Quach, Quoc-Hoang-Quyen Vo

**Affiliations:** a International University, School of Biomedical Engineering, Ho Chi Minh City, Vietnam; b Vietnam National University HCMC, Ho Chi Minh City, Vietnam

**Keywords:** human breast cancer, backscattering polarization imaging, depolarization power, Mueller matrix transformation

## Abstract

**Significance:**

Breast cancer ranks second in the world in terms of the number of women diagnosed. Effective methods for its early-stage detection are critical for facilitating timely intervention and lowering the mortality rate.

**Aim:**

Polarimetry provides much useful information on the structural properties of breast cancer tissue samples and is a valuable diagnostic tool. The present study classifies human breast tissue samples as healthy or cancerous utilizing a surface-illuminated backscatter polarization imaging technique.

**Approach:**

The viability of the proposed approach is demonstrated using 95 breast tissue samples, including 35 healthy samples, 20 benign cancer samples, 20 grade-2 malignant samples, and 20 grade-3 malignant samples.

**Results:**

The observation results reveal that element $m_{23}$ in the Mueller matrix of the healthy samples has a deeper color and greater intensity than that in the breast cancer samples. Conversely, element $m_{32}$ shows a lighter color and reduced intensity. Finally, element $m_{44}$ has a darker color in the healthy samples than in the cancer samples. The analysis of variance test results and frequency distribution histograms confirm that elements $m_{23}$, $m_{32}$, and $m_{44}$ provide an effective means of detecting and classifying human breast tissue samples.

**Conclusions:**

Overall, the results indicate that surface-illuminated backscatter polarization imaging has significant potential as an assistive tool for breast cancer diagnosis and classification.

## 1 Introduction

With an estimated 2.26 million new cases of breast cancer globally and around 685,000 breast cancer-related deaths, breast cancer accounted for around 24.5% of all cancer cases around the world in 2020 and 15.5% of cancer deaths among women.^1^^,^^2^ Breast cancer was the most common cause of cancer mortality in women in 2020 and the fifth most common cause of cancer death.^2^ Furthermore, the number of breast cancer cases is expected to exceed 4.4 million by 2070.^1^

The human breast is made up primarily of adipose (fat) and milk-producing glandular lobules.^2^ The lobules connect to create lobes, with each breast having around 15 to 20 lobes in total. Within the lobules, milk glands are connected to small ducts, which join together to form larger milk ducts that emerge through the nipple. The breast is linked to a network of blood and lymph veins, which serve to regulate the local fluid balance along with filter out harmful substances. Breast cancer metastases are formed mainly in the lymph nodes near the breast. However, the lymph veins carry lymph fluid to lymph nodes throughout the body, and thus breast cancer frequently spreads to other nearby lymph nodes, such as under the arm, above the collarbone, or in the chest. The breast is physically supported by a network of connective tissue that contains collagen. The breast density in mammograms is related to the relative amount of connective tissue,^3^ and a higher density is generally associated with a higher cancer risk.^4^

Breast cancer is defined as the uncontrolled proliferation of epithelial cells in the breast and is classified according to the cellular origin and tumor stage.^4^ Breast cancer can develop in the ducts or the lobules. The most prevalent type of breast cancer is ductal carcinoma, which develops in the cells that coat the duct walls. Breast cancer in the early stage (stage 0) is noninvasive and is referred to as being *in situ* (e.g., ductal carcinoma *in situ*). The more advanced cancer stages (I to IV) are distinguished by invasive tumors that have spread to other breast tissue, lymph nodes, and/or organs. Stages I to III are categorized based on the tumor size and presence (or otherwise) of cancer cells in the lymph nodes. In stage IV, the cancer spreads to other organs, most commonly the bones, lungs, liver, and brain, and palliative care or end-of-life care is generally required.

Although breast cancer patients have numerous treatment options available to them, particularly in the early stage, the majority of late-stage patients must undergo tumor removal surgery.^4^ Depending on the tumor size, location, disease stage, menopausal state, hormone receptor status, and cancer recurrence, surgical intervention may involve breast-conserving surgery (lumpectomy), mastectomy (complete breast removal), or lymph node removal. The surgical procedure is commonly followed by radiation therapy, hormone therapy, or chemotherapy. If the breast cancer has progressed to the bones, lungs, or brain, radiation therapy is usually delivered after breast-conserving surgery or, in rare cases, after mastectomy. Hormonal treatment, by contrast, is used to treat hormone receptor-positive breast cancer, while chemotherapy is routinely used after surgery for early stage breast cancer.

Many diagnostic imaging technologies, including mammography, magnetic resonance imaging (MRI), positron emission tomography (PET), computed tomography, and ultrasound, are available for detecting breast cancer in the early stage. Based on the imaging results, histopathology may then be performed to determine the disease.^5^ Mammography, which uses X-ray images to identify breast cancer tissue, is the most sensitive method for detecting breast cancer but has a high incidence rate of false-positive and false-negative readings, which can lead to therapeutic complications, overtreatment, and mental distress.^6^ MRI can detect minute abnormalities that may be missed by mammography; however, it is expensive and has low specificity, resulting in possible overdiagnosis.^7^ PET is the most accurate method for visualizing the progression of cancer and its treatment response. However, it requires the injection of radioactive tracer, which can cause numerous side effects and radiation exposure.^8^

On some occasions, imaging techniques may yield false-positive readings, which prompt the physician to conduct a biopsy, only to obtain a benign pathologic result. This not only incurs unnecessary medical expense but also causes the patient needless pain and mental suffering. Thus, high sensitivity and accurate diagnostic imaging techniques are required to support the physician in deciding whether a biopsy procedure is required.^9^

Polarimetry is a well-established technique for studying matter properties using polarized light.^10^^,^^11^ Various levels of information can be obtained, depending on the specific configuration of samples and number of polarization measurements taken.^12^ Images with concentrated contrast in superficial skin layers, for example, can be obtained by taking just two measurements with a polarizer before and after the sample (one with the polarization axes aligned and one with them crossed).^13^ A thorough description of a sample’s polarization properties, on the other hand, necessitates numerous (at least 16) polarization-sensitive measurements. The Mueller matrix constructed in polarimetry techniques relates the input and output polarization states and contains 16 elements, which between them completely describe how a sample interacts with all types of polarized light.^14^^,^^15^ The polarization state of a polarized light beam changes when it interacts with the sample, and thus analyzing the state of polarization of the emergent light yields valuable information regarding the optical and structural properties of the sample.^16^ Mueller matrix polarimetry (MMP) technique has numerous advantages for biomedical diagnostics.^15^^,^^17^ For example, the Mueller matrix provides the picture contrast of the surface tissue layers by up to 85% and thus the information for early detection of precancerous alterations are figured.^18^^,^^19^ Furthermore, the Mueller matrix provides critical information on the architecture of abnormal tissue areas^19^^–^^21^ and can be used in conjunction with a wide variety of optical devices, such as microscopes and endoscopes, by adding the appropriate polarization components.^22^^,^^23^ For instance, numerous studies have demonstrated the feasibility of detecting early stage cancer by combining MMP with endoscopy.^23^^,^^24^

The present study analyzes the polarization features of human breast cancer tissue utilizing a polarimetric imaging system based on a charge-coupled device (CCD) camera.^25^^–^^30^ In the proposed approach, the backscattered light from the breast tissue is captured by the camera and processed by the Stokes–Mueller method to determine the Mueller matrix image and corresponding optical properties of the sample. The sample is then classified into one of four different classes, namely normal, cancer benign, or cancer malignant (grade 2 or 3) based on an analysis of the average intensity of the Mueller matrix elements, the analysis of variance (ANOVA) test results, and the frequency distribution histograms (FDHs) of the pixel intensity.

## 2 Methodology

### 2.1 Sample Preparation

**Fig. 1:**
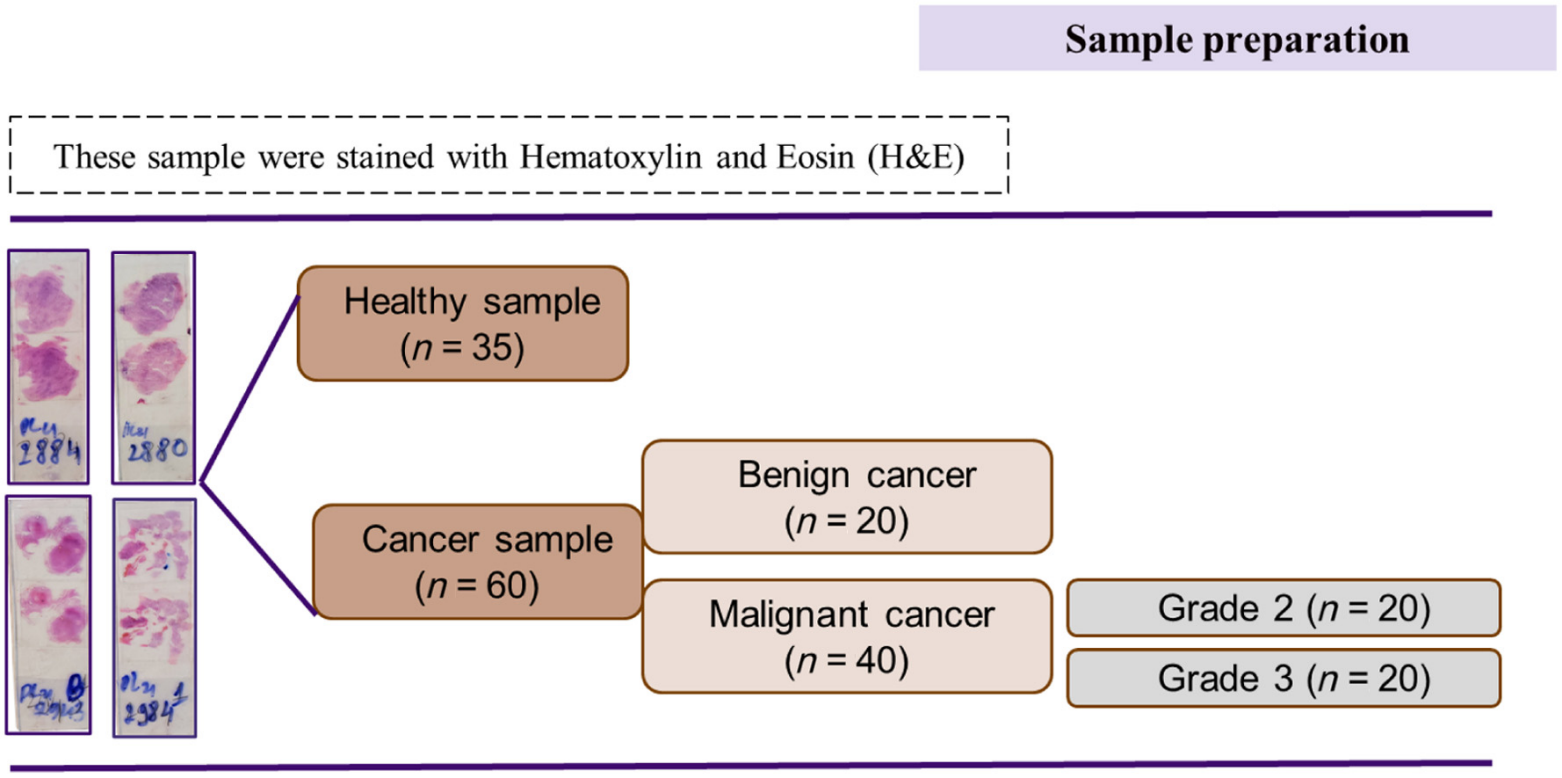
Sample preparation processing.

As shown in Fig. 1, 95 breast tissue samples were obtained, including 35 healthy samples and 60 cancer samples. The cancer samples comprised 20 benign samples, 20 grade-2 malignant samples, and 20 grade-3 malignant samples. Each sample consisted of two to three slices of tissue attached to a microscope slide. The samples were acquired from a specialist clinic at the Ho Chi Minh City Oncology Hospital in Vietnam with the commitment that the samples would be used only for scientific research. All of the preparation-related excision, tissue processing, attachment, staining, registration, and histopathological analysis procedures were performed by qualified pathologists before transferring the samples for experiment. The tissues were sliced with a thickness of 5  μm and were stained with hematoxylin and eosin. Each slice was measured three to five times using the polarization imaging system, and the average intensity values were then calculated.

### 2.2 Experimental Setup

**Fig. 2:**
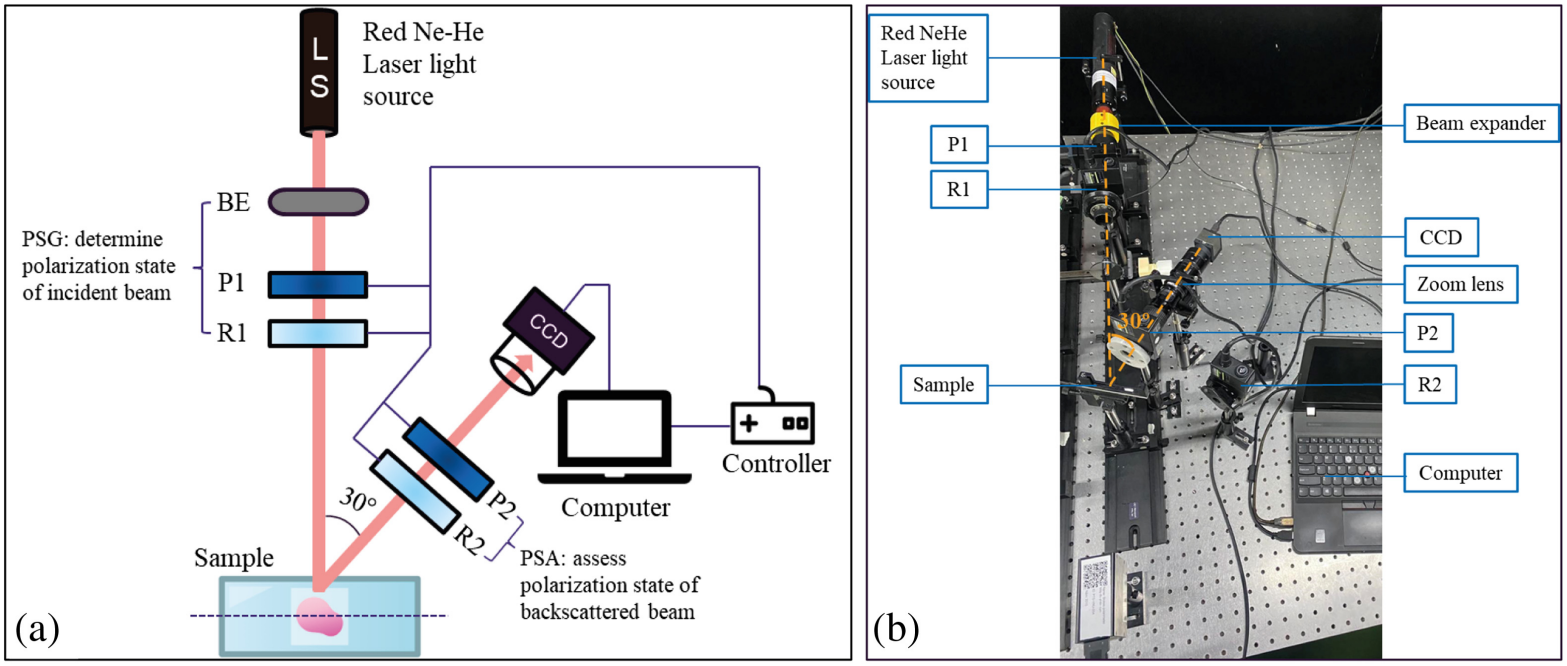
(a) Schematic illustration of experimental setup, and (b) photograph of experimental arrangement (LS, laser source; P1–P2, linear polarizers (operated by stepping motors: 0 deg, 45 deg, 90 deg, and 135 deg); R1–R2, quarter-wave plates (RH 45 deg, LH – 45 deg); CCD, CCD camera; BE, beam expander).

Figure 2 illustrates the backscattering Mueller matrix polarization imaging system constructed in the present study. As shown, the system comprised two major components: a polarization state generator (PSG) and a polarization state analyzer (PSA). The PSG block produced the desired polarization state of the incident beam, whereas the PSA block examined the polarization state of the sample-affected beam. The generator consisted of a stabilized red He-Ne laser light source (LS, HNLS008R, Thorlabs, Inc.), a quarter-wave plate polarizer (R1, QWP0-63304-4-R10, CVI Co.) controlled by a motor-driven stage (SGSP-60YAW-0B, Sigma Koki Co.), a linear polarizer (P1, GTH5M, Thorlabs Co.) controlled by a second motor-driven stage, and a beam expander (BE). The BE increased the diameter of the laser beam from 0.5 to 1.5 cm, thereby enabling more coverage the sample to improve the accuracy and quality of the results. The analyzer consisted of a linear polarizer (P2, GTH5M, Thorlabs, Inc.), a quarter-wave plate (R2, QWP0-63304-4-R10, CVI Co.), and a CCD camera (CCD, DCU224C, Thorlabs, Inc.) fitted with a 6.5× zoom lens and connected to a computer for image acquisition and analysis purposes.

In the measurement process, the light from the laser source was passed through the BE, quarter-wave plate (R1), and linear polarizer (P1) of the generator block to produce six different polarization states: horizontal linear (H), vertical linear (V), 45-deg linear (P), 135-deg linear (M), right-hand circular (R), and left-hand circular (L). The linear states were produced by rotating the polarizer (P1) and sliding the quarter-wave plate (R1) out of the light path, while the circular polarization states were generated by just rotating R1. The polarized light was focused on the sample surface. The back-scattered light passed through the quarter-wave plate (R2) and linear polarizer (P2) and was captured by the CCD camera. The captured images were then interfaced to the computer for further processing using the Stokes–Mueller method.

In establishing the experimental setup, the linear polarizers (P1 and P2) and quarter-wave plates (R1 and R2) were individually mounted on stepping motors to minimize rotation errors. Moreover, to minimize light reflection effects from the sample surface, the angle between the generator axis and analyzer axis was set as 30 deg. For the calibration process, a standard mirror (BB1-E02, Thorlabs, Inc.) was captured and calculated the Mueller matrix images with an accuracy of $10^{-2}$ (see Ref. 30). After calibration, the polarimeter system was conducted the experiments.

### 2.3 Mueller Matrix Imaging

**Fig. 3:**
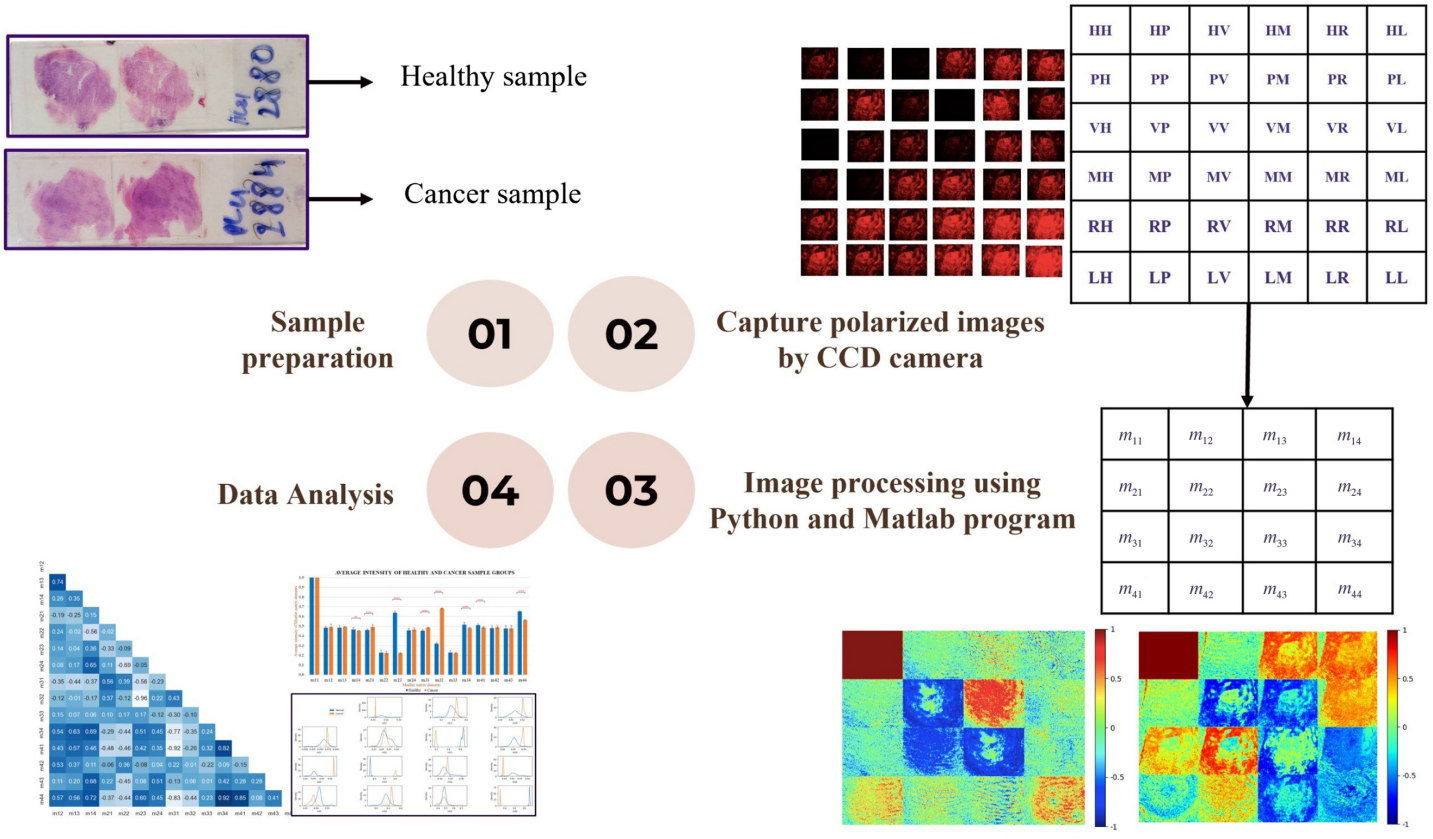
Experimental procedure.

Figure 3 shows the experimental procedure of the proposed study with four stages, including sample preparation, capturing 36 polarization images, image processing and calculation of Mueller matrix elements and polarization parameters, and then data analysis. In the Mueller matrix imaging technique, the input and output Stoke vectors obtained for the six polarization states are replaced by 36 images showing the corresponding states of polarization.^17^ The Mueller matrix image for the sample is then computed as shown in Eq. (1), where H, P, V, and M refer to linear polarized lights with orientations of 0 deg, 45 deg, 90 deg, and 135 deg, respectively. And L and R correspond to left-handed and right-handed circular polarization lights, respectively. Thus, element $m_{11}$, for example, requires four measurements, HH, VV, HV, and VH since $m_{11}=\mathrm{HH}+\mathrm{HV}+\mathrm{VH}+\mathrm{VV}$. In the present study, the matrix elements were constructed using a self-written program coded in Python to merge the individual polarized images as required

$$M=[\begin{array}{cccc} m_{11} & m_{12} & m_{13} & m_{14}\\ m_{21} & m_{22} & m_{23} & m_{24}\\ m_{31} & m_{32} & m_{33} & m_{34}\\ m_{41} & m_{42} & m_{43} & m_{44} \end{array}]\;=[\begin{array}{cccc} \mathrm{HH}+\mathrm{HV}+\mathrm{VH}+\mathrm{VV} & \mathrm{HH}+\mathrm{HV}-\mathrm{VH}-\mathrm{VV} & \mathrm{PH}+\mathrm{PV}-\mathrm{MH}-\mathrm{MV} & \mathrm{RH}+\mathrm{RV}-\mathrm{LH}-\mathrm{LV}\\ \mathrm{HH}-\mathrm{HV}+\mathrm{VH}-\mathrm{VV} & \mathrm{HH}-\mathrm{HV}-\mathrm{VH}+\mathrm{VV} & \mathrm{PH}-\mathrm{PV}-\mathrm{MH}+\mathrm{MV} & \mathrm{RH}-\mathrm{RV}-\mathrm{LH}+\mathrm{LV}\\ \mathrm{HP}-\mathrm{HM}+\mathrm{VP}-\mathrm{VM} & \mathrm{HP}-\mathrm{HM}-\mathrm{VP}+\mathrm{VM} & \mathrm{PP}-\mathrm{PM}-\mathrm{MP}+\mathrm{MM} & \mathrm{RP}-\mathrm{RM}-\mathrm{LP}+\mathrm{LM}\\ \mathrm{HR}-\mathrm{HL}+\mathrm{VR}-\mathrm{VL} & \mathrm{HR}-\mathrm{HL}-\mathrm{VR}+\mathrm{VL} & \mathrm{PR}-\mathrm{PL}-\mathrm{MR}+\mathrm{ML} & \mathrm{RR}-\mathrm{RL}-\mathrm{LR}+\mathrm{LL} \end{array}].$$ (1)

### 2.4 Mueller Matrix Transformation Parameters

Mueller matrix transformation (MMT) techniques provide a quantitative approach for characterizing the Mueller matrix elements in such a way as to evaluate specific properties of the sample, including the microstructure, density and size of the subwavelength scatterers, orientation and alignment of the fibers, presence of scatterers of various shapes and sizes, and so forth.^31^^,^^32^ Various MMT parameters are available,^33^ depending on the particular characteristics of interest. For example, He et al.^34^ proposed the following four parameters for characterizing the polarization properties of biological tissue samples:

$$A=\frac{2(m_{22}+m_{33})\sqrt{(m_{22}-m_{33}{)}^{2}+(m_{23}+m_{32}{)}^{2}}}{(m_{22}+m_{33}{)}^{2}+(m_{22}-m_{33}{)}^{2}+(m_{23}+m_{32}{)}^{2}},$$ (2)

$$b=\frac{m_{22}+m_{33}}{2},$$ (3)

$$t=\frac{\sqrt{(m_{22}-m_{33}{)}^{2}+(m_{23}+m_{32}{)}^{2}}}{2},$$ (4)

$$\Delta =1-\frac{\left|m_{22}|+|m_{33}|+|m_{44}\right|}{a}0\leq \Delta \leq 1,$$ (5)

where A is the anisotropy index, b is the depolarization power factor, t is an index related to the magnitude of the anisotropy, and Δ is the depolarization power.

## 3 Results

### 3.1 Mueller Matrix of Healthy Human Breast Samples

**Fig. 4:**
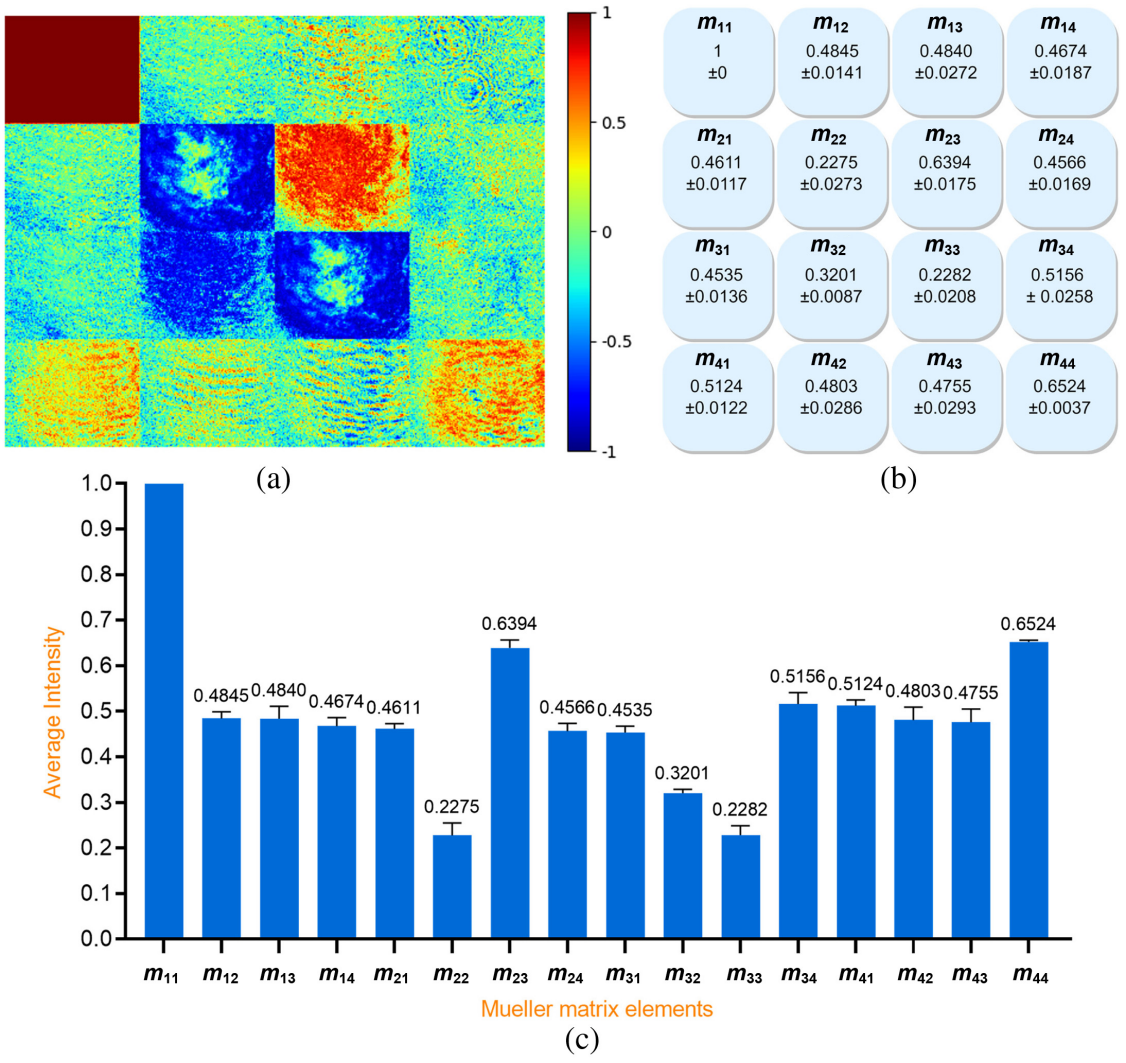
(a) Normalized Mueller matrix image of typical healthy human breast sample, (b) standard deviation values, and (c) average intensity values of 210 Mueller matrix images of healthy human breast tissue samples.

A total of 210 Mueller matrix images were obtained for the 35 healthy breast samples. Figure 4(a) shows the normalized Mueller matrix image of a typical healthy human breast sample. The color bar indicates the colorimetric intensity of the matrix elements in the interval of [−1,1]. As noted above, element $m_{11}$ is used for normalization purposes and thus has the maximum value of 1 (red). The average intensity of each Mueller matrix element was computed and then normalized to a value in the range of −1 to 1 using the intensity of element $m_{11}$ as a reference. The corresponding results for the standard deviation and average values of the matrix elements are presented in Figs. 4(b) and 4(c), respectively.

### 3.2 Mueller Matrix of Human Breast Cancer Samples

#### 3.2.1 Benign human breast cancer sample

**Fig. 5:**
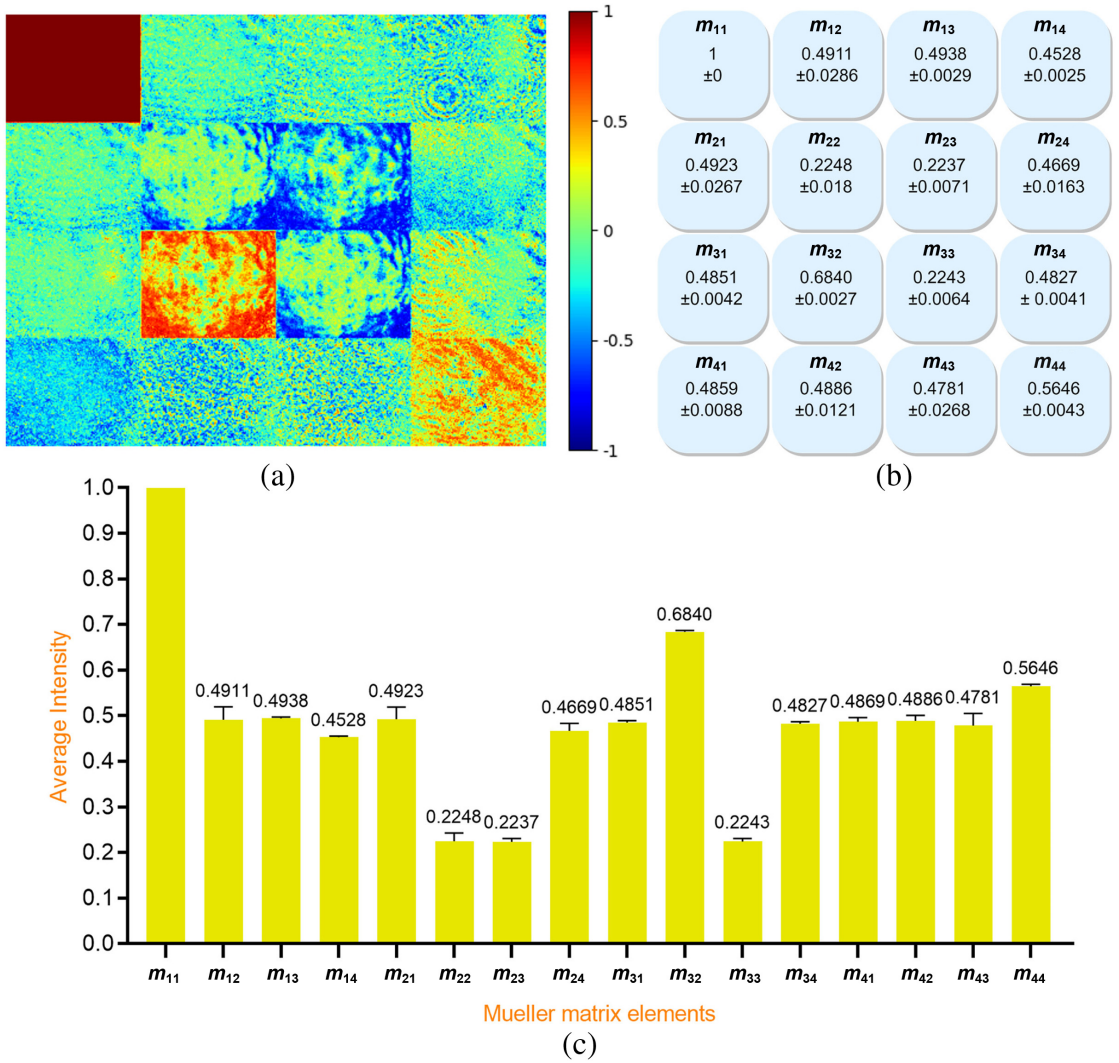
(a) Normalized Mueller matrix image of typical benign human breast cancer sample, (b) standard deviation values, and (c) average intensity values of 120 Mueller matrix images of benign human breast cancer samples.

A total of 120 Mueller matrix images were obtained of the 20 benign breast cancer samples. Figure 5(a) shows the Mueller matrix image of a typical sample in the benign breast cancer sample set, while Figs. 5(b) and 5(c) show the standard deviation and mean values of the intensities of the 16 elements in each image.

#### 3.2.2 Grade-2 malignant human breast cancer sample

**Fig. 6:**
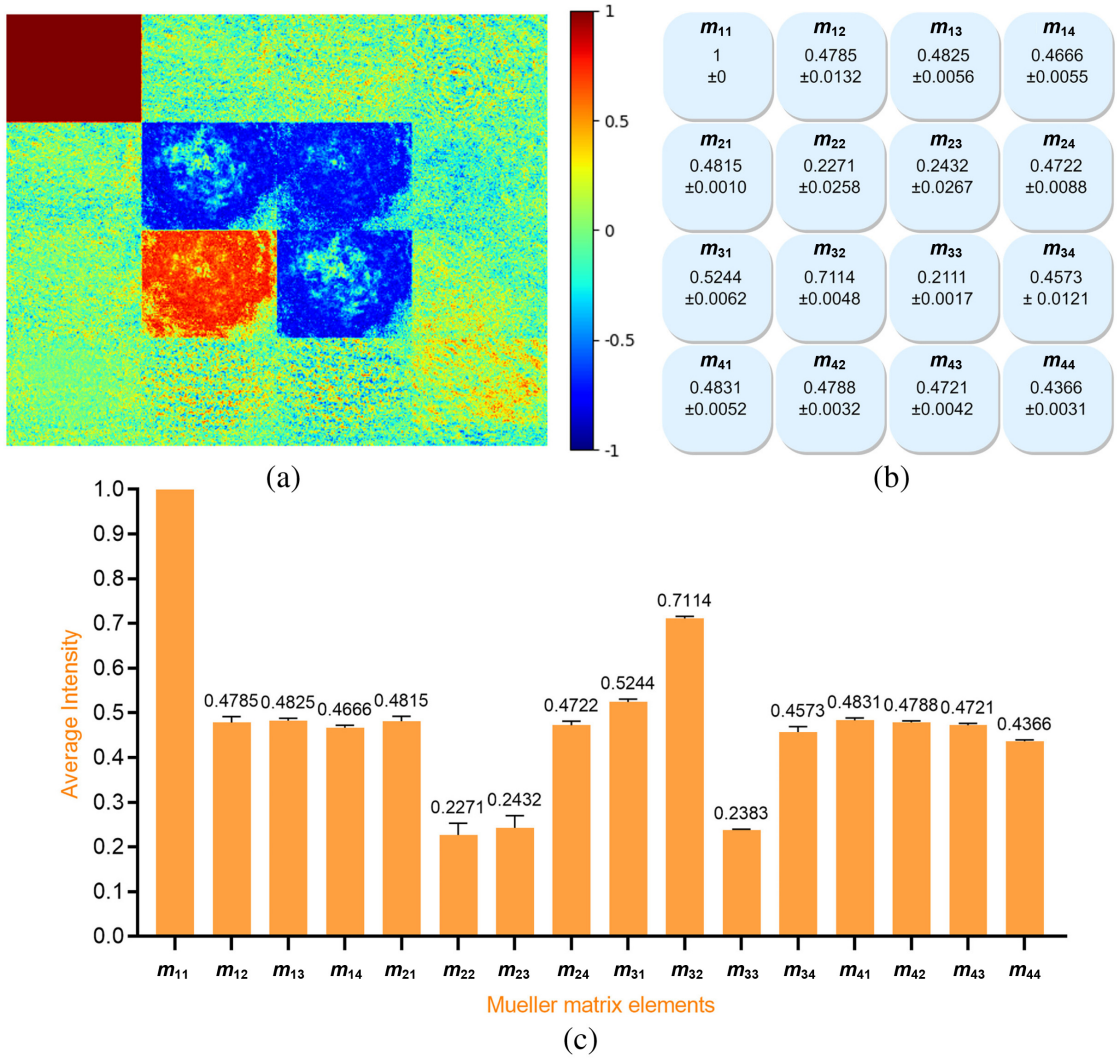
(a) Normalized Mueller matrix image of typical grade-2 malignant human breast cancer sample, (b) standard deviation values, and (c) average intensity values of 120 Mueller matrix images of grade-2 malignant human breast cancer samples.

A total of 120 Mueller matrix images were obtained of the 20 grade-2 malignant human breast cancer samples. Figure 6(a) presents the Mueller matrix image of a typical grade-2 malignant sample, while Figs. 6(b) and 6(c) show the standard deviation and mean values of the Mueller matrix element intensities for the 20 samples.

#### 3.2.3 Mueller matrix of grade-3 malignant human breast cancer sample

**Fig. 7:**
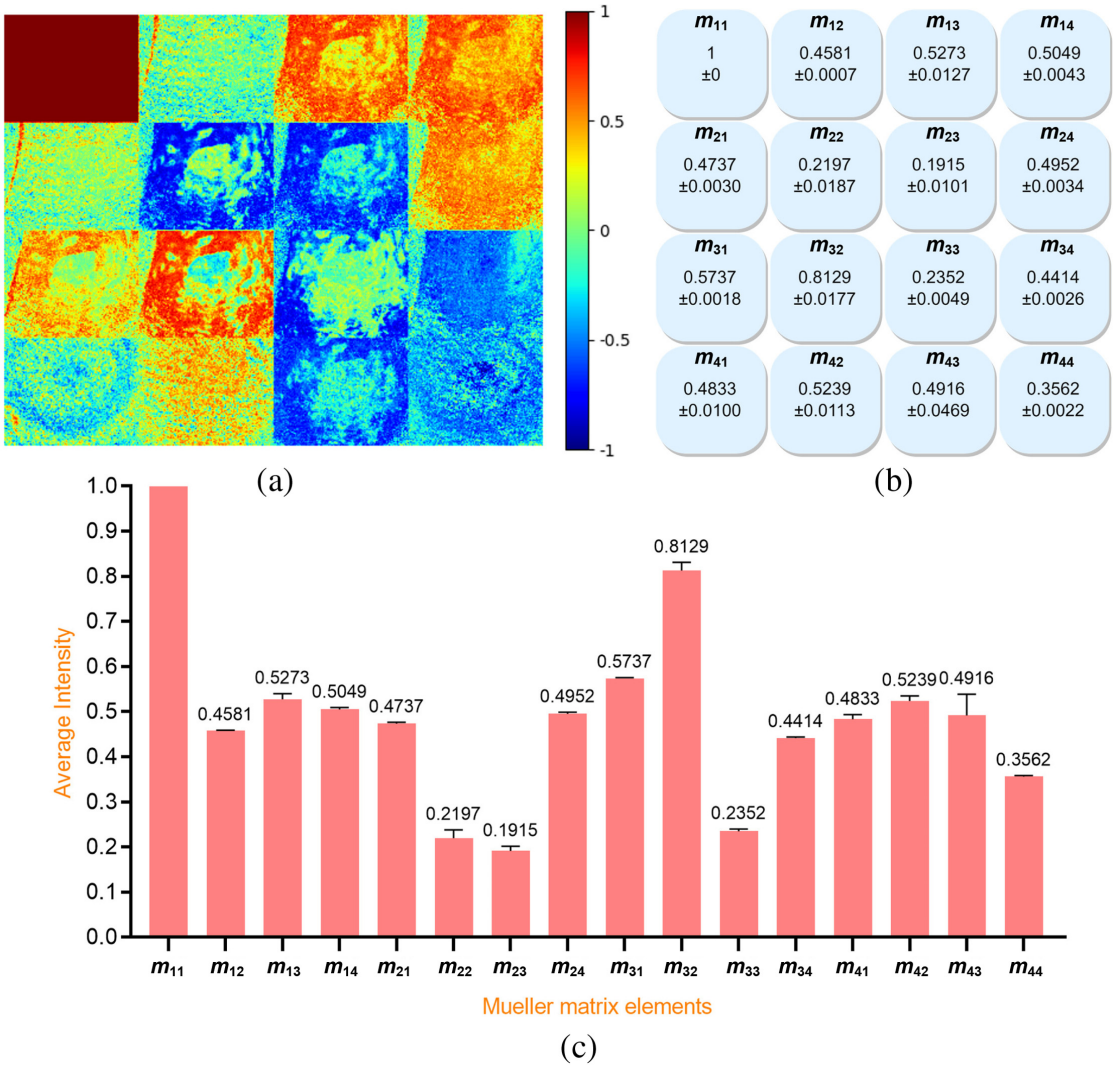
(a) Normalized Mueller matrix image of typical grade-3 malignant human breast cancer sample, (b) standard deviation values, and (c) average intensity values of 120 Mueller matrix images of grade-3 malignant human breast cancer samples.

A total of 120 Mueller matrix images were similarly obtained of the 20 grade-3 malignant human breast cancer samples. Figure 7(a) shows the normalized Mueller matrix image of a typical sample, while Figs. 7(b) and 7(c) show the mean and standard deviation values of the Mueller matrix element intensities for the 20 samples.

## 4 Discussion

### 4.1 Mueller Matrix Analysis

#### 4.1.1 Comparison between healthy and cancerous human breast tissue samples

**Fig. 8:**
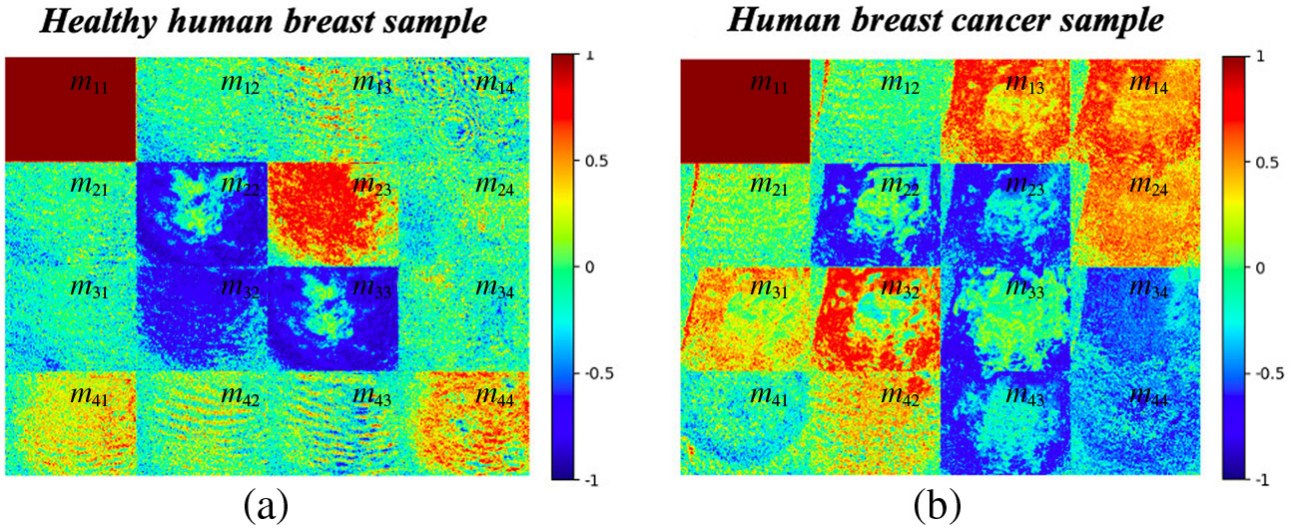
Normalized Mueller matrix images of (a) healthy human breast sample and (b) cancerous sample.

Figure 8 compares the Mueller matrix images of a healthy breast tissue sample (left) and cancerous breast tissue sample (right). The Mueller matrix image of the cancerous sample generally shows more color change than the healthy sample and thus provides a rough guideline for estimating the pathological state of the sample as either healthy or cancerous. A detailed inspection shows that the intensity difference between the two samples is particularly pronounced for elements $m_{23},m_{32}$, and $m_{44}$. For example, the color intensity of element $m_{23}$ for the healthy sample is substantially brighter (more than 0.5) than that for the corresponding element in the cancerous sample (see also Table 1). By contrast, for element $m_{32}$, the color intensity of the cancer sample is much higher than that of the healthy sample. Finally, for element $m_{44}$, the color intensity of the healthy sample is once again darker than that of the cancer samples.

**Table 1:** Average intensity values of 16 elements in normalized Mueller matrix images of healthy and cancerous human breast tissue samples.

Average intensity	Healthy	Cancer	Percentage difference (%)
$m_{11}$	1 ± 0	1 ± 0	0
$m_{12}$	0.4845 ± 0.0141	0.4581 ± 0.0007	5.4
$m_{13}$	0.4840 ± 0.0272	0.5273 ± 0.0127	8.2
$m_{14}$	0.4674 ± 0.0187	0.5049 ± 0.0043	7.4
$m_{21}$	0.4611 ± 0.0117	0.4737 ± 0.0030	2.7
$m_{22}$	0.2275 ± 0.0273	0.2197 ± 0.0187	3.4
$m_{23}$	0.6394 ± 0.0175	0.1915 ± 0.0101	70.1
$m_{24}$	0.4566 ± 0.0169	0.4952 ± 0.0034	7.8
$m_{31}$	0.4535 ± 0.0136	0.5737 ± 0.0018	21.0
$m_{32}$	0.3201 ± 0.0087	0.8129 ± 0.0177	60.6
$m_{33}$	0.2282 ± 0.0208	0.2052 ± 0.0049	10.1
$m_{34}$	0.5156 ± 0.0258	0.4414 ± 0.0026	14.4
$m_{41}$	0.5124 ± 0.0122	0.4833 ± 0.0100	5.7
$m_{42}$	0.4803 ± 0.0286	0.5239 ± 0.0113	8.3
$m_{43}$	0.4755 ± 0.0293	0.4916 ± 0.0469	3.3
$m_{44}$	0.6524 ± 0.0037	0.3562 ± 0.0022	45.4

**Fig. 9:**
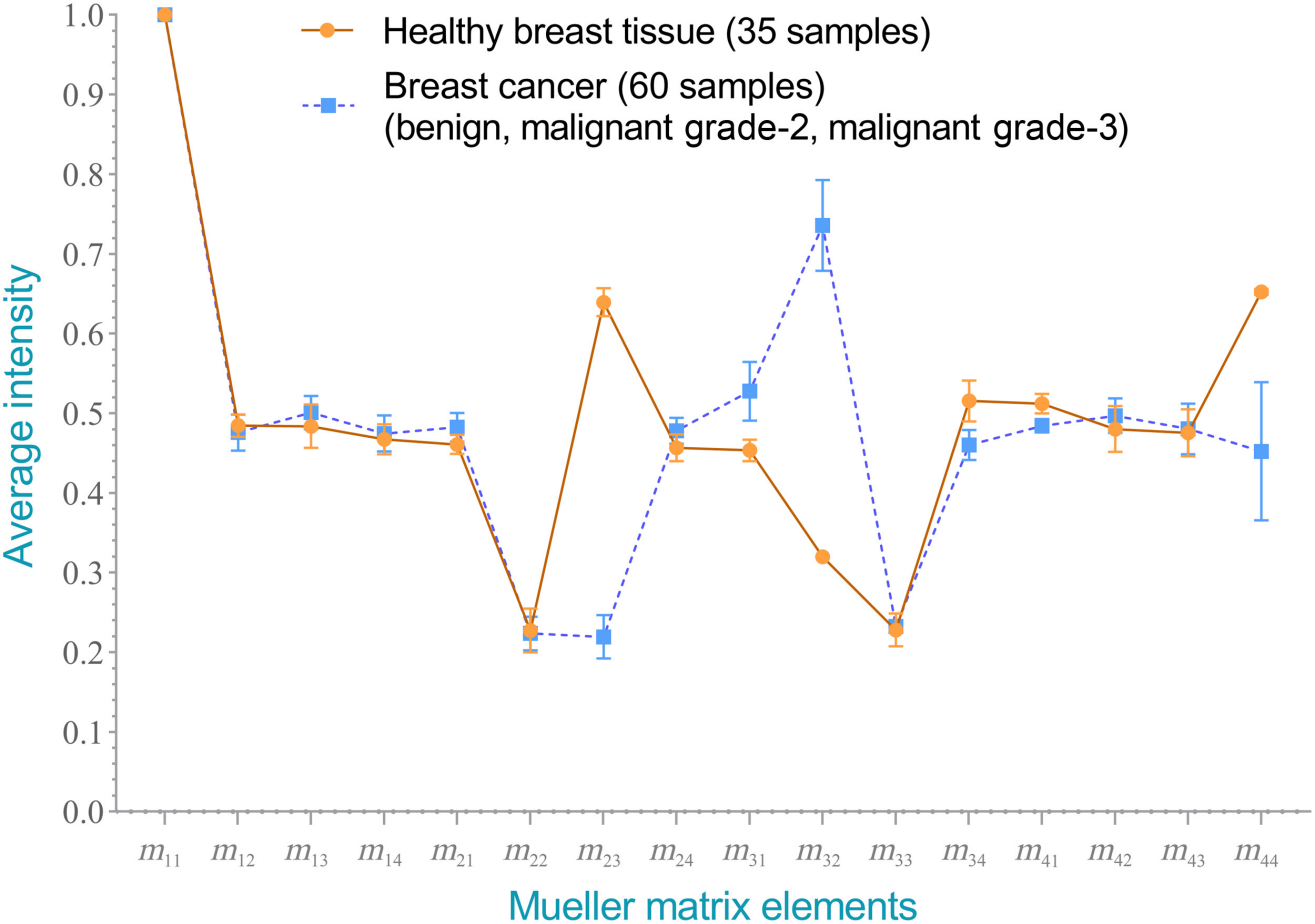
Average normalized intensity of each element in Mueller matrixes of healthy and cancerous human breast tissue samples.

**Fig. 10:**
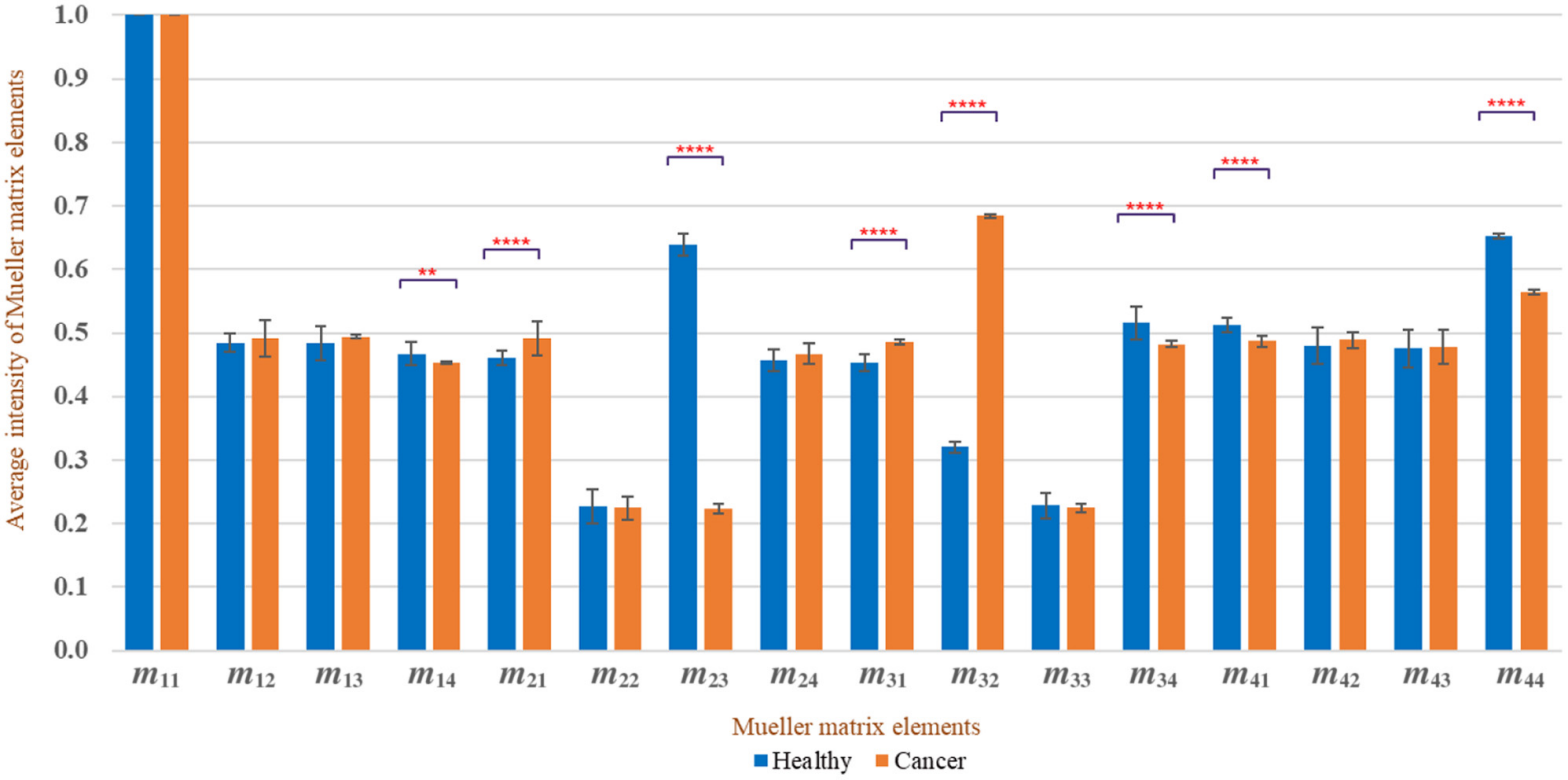
Average normalized intensities of each element in Mueller matrixes of healthy and cancerous human breast tissue samples. The star symbols represent the p-values: 0.002 (**), <0.0001 (****), as determined by a paired t-test.

Figures 9 and 10 show the average intensities of the 16 elements in the Mueller matrixes of the healthy and cancerous breast tissue samples. Clear differences are observed between the intensities of the $m_{23},m_{32}$, and $m_{44}$ elements in the two samples. In particular, the intensity of the $m_{23}$ element in the cancer sample (0.1915) is substantially lower than that in the healthy sample (0.6394). Conversely, the intensity of element $m_{32}$ in the cancer group (0.8129) is around three times higher than that in the healthy group (0.320). Finally, the intensity of element $m_{44}$ in the cancer sample (0.3562) is around half that of the intensity in the healthy sample (0.6524). A paired t-test comparison revealed that the difference in the intensity values between the two different samples was significant for each of the three elements (p<0.00001).

As shown in Fig. 10, elements $m_{14},m_{21},m_{31},m_{34}$, and $m_{41}$ also show a substantial (but smaller) difference in intensity between the samples of different classes (p<0.0001). Thus, overall, the results confirm that the intensity of the Mueller matrix elements provides a feasible means of distinguishing between healthy and cancerous human breast tissue samples.

**Fig. 11:**
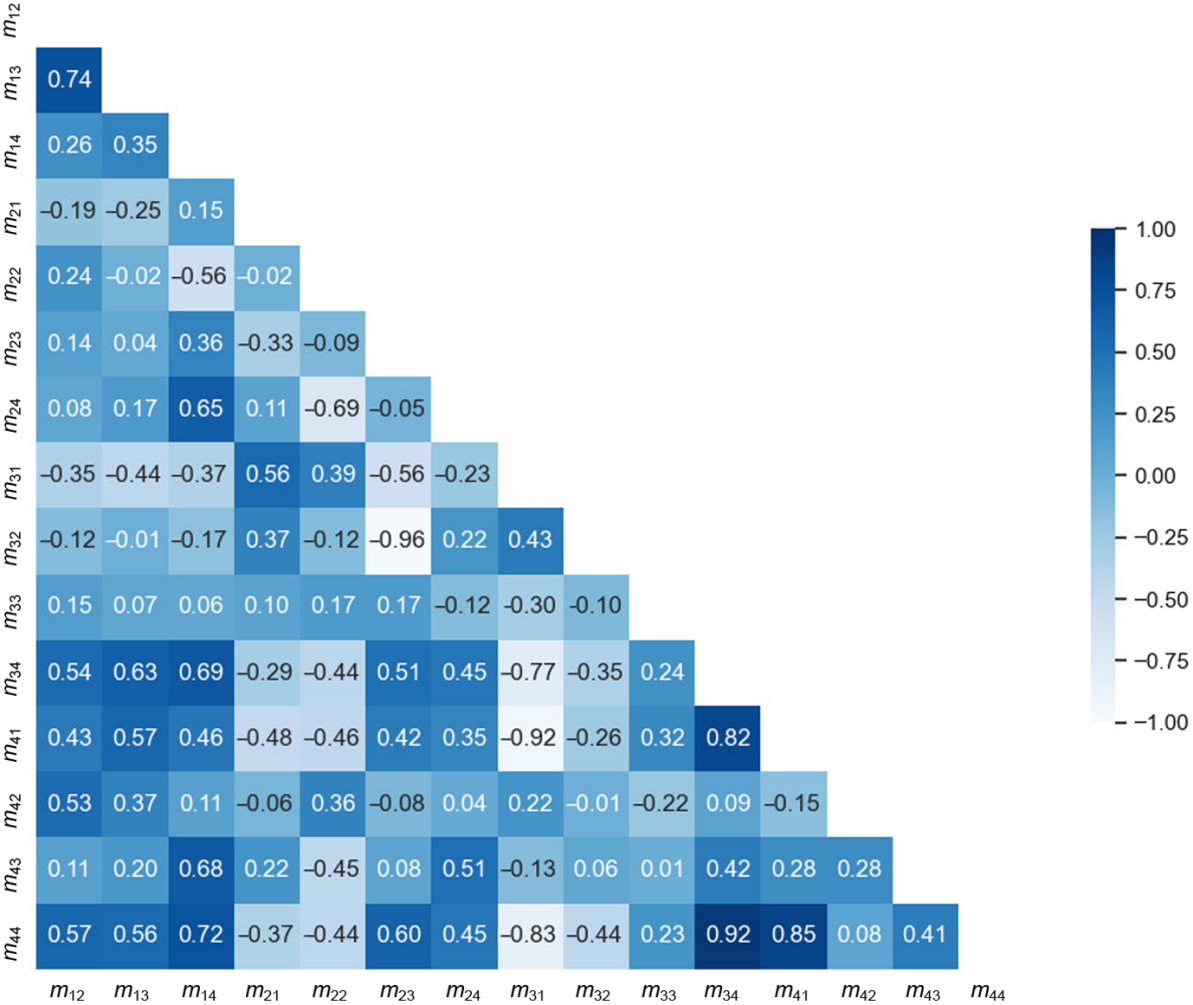
Correlation matrix of Mueller matrix element intensities for healthy and cancerous human breast tissue samples.

Figure 11 shows the correlation coefficients between each pair of elements in the Mueller matrixes of the two sample classes, demonstrating the paired relationship between 15 variables in the correlation matrix. When the correlation coefficient value is positive and has a large value (close to 1), it implies the existence of a similar and consistent relationship between the two variables. Conversely, if the correlation coefficient value is negative and has a small value (close to −1), it indicates the discrepancy between the pair of variables. As shown, the correlation coefficient has a relatively low value (<0.80) for all of the element pairs other than those between $m_{44}$ and $m_{34}$ and $m_{41}$, respectively, and $m_{41}$ and $m_{34}$. In other words, the results confirm that the intensity values of elements $m_{23}$, $m_{32}$, and $m_{44}$ enable healthy and cancerous breast tissue samples to be reliably differentiated.^35^

#### 4.1.2 Comparison among healthy, benign, and malignant (grades 2 and 3) human breast cancer samples

**Fig. 12:**
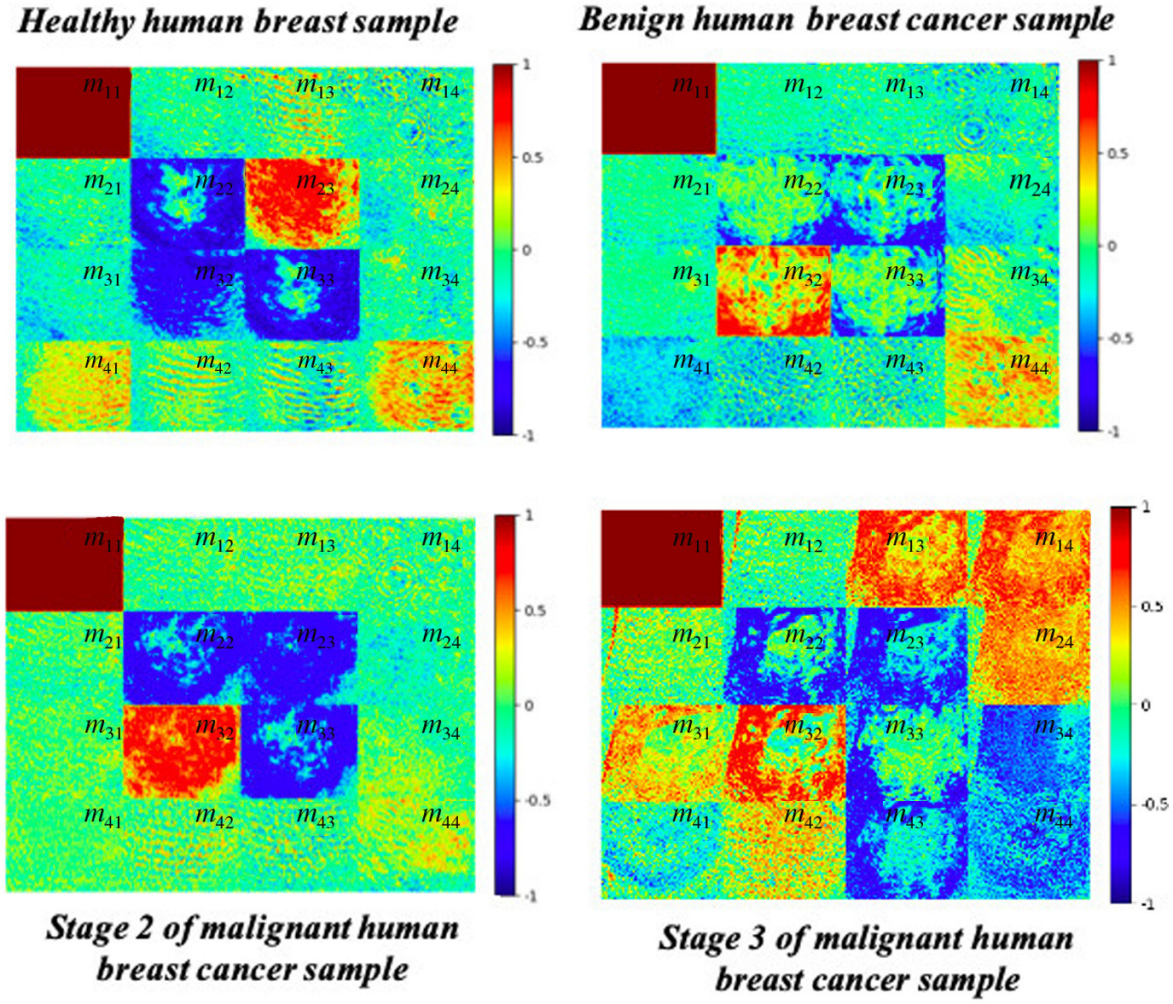
Normalized Mueller matrix images of healthy and cancerous human breast tissue samples.

**Fig. 13:**
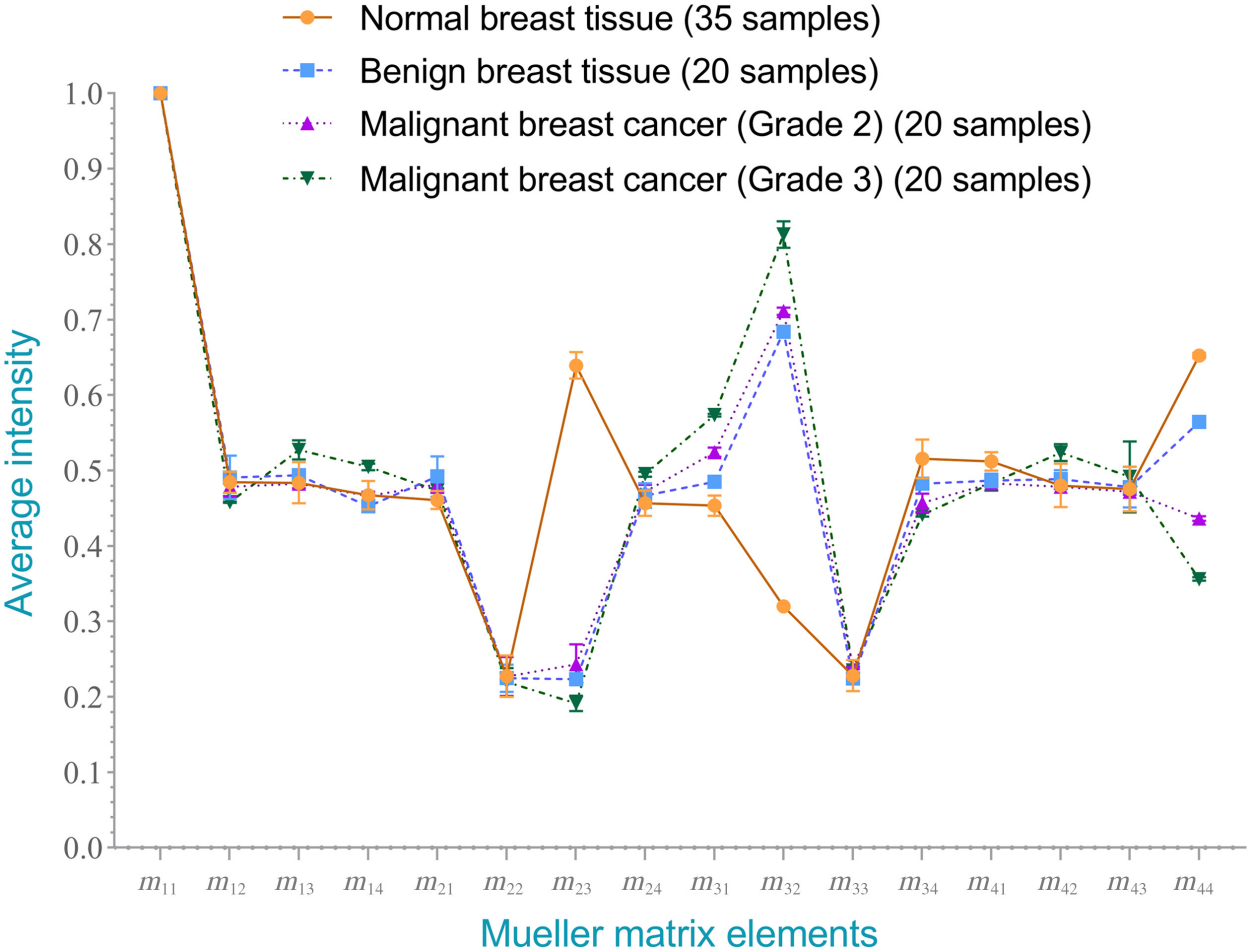
Average normalized intensity of each element in Mueller matrixes of healthy and cancerous human breast tissue samples.

Figure 12 compares the Mueller matrix images of the four sample types of a randomly selected sample set (namely, healthy, benign, grade-2 malignant, and grade-3 malignant). It is noted that, a set of 210 Mueller matrix images was obtained from the 35 healthy breast samples, each set of 120 Mueller matrix images was obtained from the 20 benign breast cancer samples, grade-2, and grade-3 malignant human breast cancer samples, respectively. For statistics of all measurement sample groups, the average intensity values and their standard deviations of four sample group were shown in Table 2 and Fig. 13. Obvious color intensity differences are observed between the four samples, particularly for elements $m_{23},m_{32}$, and $m_{44}$. The intensity of element $m_{23}$ in the healthy sample is greater (i.e., darker) than that of the corresponding element in any of the cancer samples. Thus, the intensity of this element provides a quick and simple approach for screening potential cancerous samples for further histopathological analysis. Element $m_{32}$ has a strong dark intensity in all three cancer samples (benign, grade-2, and grade-3). By contrast, it has a color intensity of <0.4 in the healthy sample. Consequently, the intensity of element $m_{32}$ also provides a quick approach for identifying cancerous samples. Finally, the intensity of element $m_{44}$ in the healthy sample has the highest value (>0.6) of all the samples. Furthermore, for the cancer samples, the intensity reduces with increasing severity. Thus, the intensity of element $m_{44}$ not only provides the means to differentiate between healthy and cancerous human breast tissue samples but also to estimate the possible grade of the cancerous samples.

**Table 2:** Average intensity values of 16 elements in normalized Mueller matrix images of healthy and cancerous human breast samples.

Average intensity	Healthy	Benign cancer	Malignant cancer (grade 2)	Malignant cancer (grade 3)
$m_{11}$	1 ± 0	1 ± 0	1 ± 0	1 ± 0
$m_{12}$	0.4845 ± 0.0141	0.4911 ± 0.0286	0.4785 ± 0.0132	0.4581 ± 0.0007
$m_{13}$	0.4840 ± 0.0272	0.4938 ± 0.0029	0.4825 ± 0.0056	0.5273 ± 0.0127
$m_{14}$	0.4674 ± 0.0187	0.4528 ± 0.0025	0.4666 ± 0.0055	0.5049 ± 0.0043
$m_{21}$	0.4611 ± 0.0117	0.4923 ± 0.0267	0.4815 ± 0.0010	0.4737 ± 0.0030
$m_{22}$	0.2275 ± 0.0273	0.2248 ± 0.018	0.2271 ± 0.0258	0.2197 ± 0.0187
$m_{23}$	0.6394 ± 0.0175	0.2237 ± 0.0071	0.2432 ± 0.0267	0.1915 ± 0.0101
$m_{24}$	0.4566 ± 0.0169	0.4669 ± 0.0163	0.4722 ± 0.0088	0.4952 ± 0.0034
$m_{31}$	0.4535 ± 0.0136	0.4851± 0.0042	0.5244 ± 0.0062	0.5737 ± 0.0018
$m_{32}$	0.3201 ± 0.0087	0.6840 ± 0.0027	0.7114 ± 0.0048	0.8129 ± 0.0177
$m_{33}$	0.2282 ± 0.0208	0.2243 ± 0.0064	0.2111 ± 0.0017	0.2052 ± 0.0049
$m_{34}$	0.5156 ± 0.0258	0.4827 ± 0.0041	0.4573 ± 0.0121	0.4414 ± 0.0026
$m_{41}$	0.5124 ± 0.0122	0.4859 ± 0.0088	0.4831 ± 0.0052	0.4833 ± 0.0100
$m_{42}$	0.4803 ± 0.0286	0.4886 ± 0.0121	0.4788 ± 0.0032	0.5239 ± 0.0113
$m_{43}$	0.4755 ± 0.0293	0.4781 ± 0.0268	0.4721 ± 0.0042	0.4916 ± 0.0469
$m_{44}$	0.6524 ± 0.0037	0.5646 ± 0.0043	0.4366 ± 0.0031	0.3562 ± 0.0022

Table 2 and Fig. 13 show the average intensities and standard deviations of the 16 elements in each of the four sample groups (namely, healthy, benign, malignant grade-2, and malignant grade-3). It is noted that the average intensities were calculated based on the values of pixels in the image of each Mueller matrix element and then normalized from the range of [0, 255] to [−1,1] accordingly. The results confirm that the main difference between the intensities of the various classes lies in elements $m_{23},m_{32}$, and $m_{44}$. For example, element $m_{23}$ in the healthy sample has a value of 0.6394 and is substantially higher than that in the benign sample (0.2237) or malignant cancer samples (grade-2: 0.2432 and grade-3: 0.1915). For element $m_{32}$, the intensity in the healthy sample (0.3201) is much lower than that in any of the cancer samples (benign: 0.6840, grade-2: 0.7114, and grade-3: 0.8129). Finally, for element *m*_44,_ the healthy sample shows the highest intensity value (0.6524), followed by the benign sample (0.5646), grade-2 malignant sample (0.4366), and grade-3 malignant sample ($m_{44}=0.3562$), respectively. Overall, the intensity values of elements *m*_23_ and *m*_44_ are arranged in descending order as follows: healthy > benign > grade-2 malignant > grade-3 malignant. Conversely, the intensity values of element *m*_32_ are arranged as follows: grade-3 malignant > grade-2 malignant > benign > healthy.

**Fig. 14:**
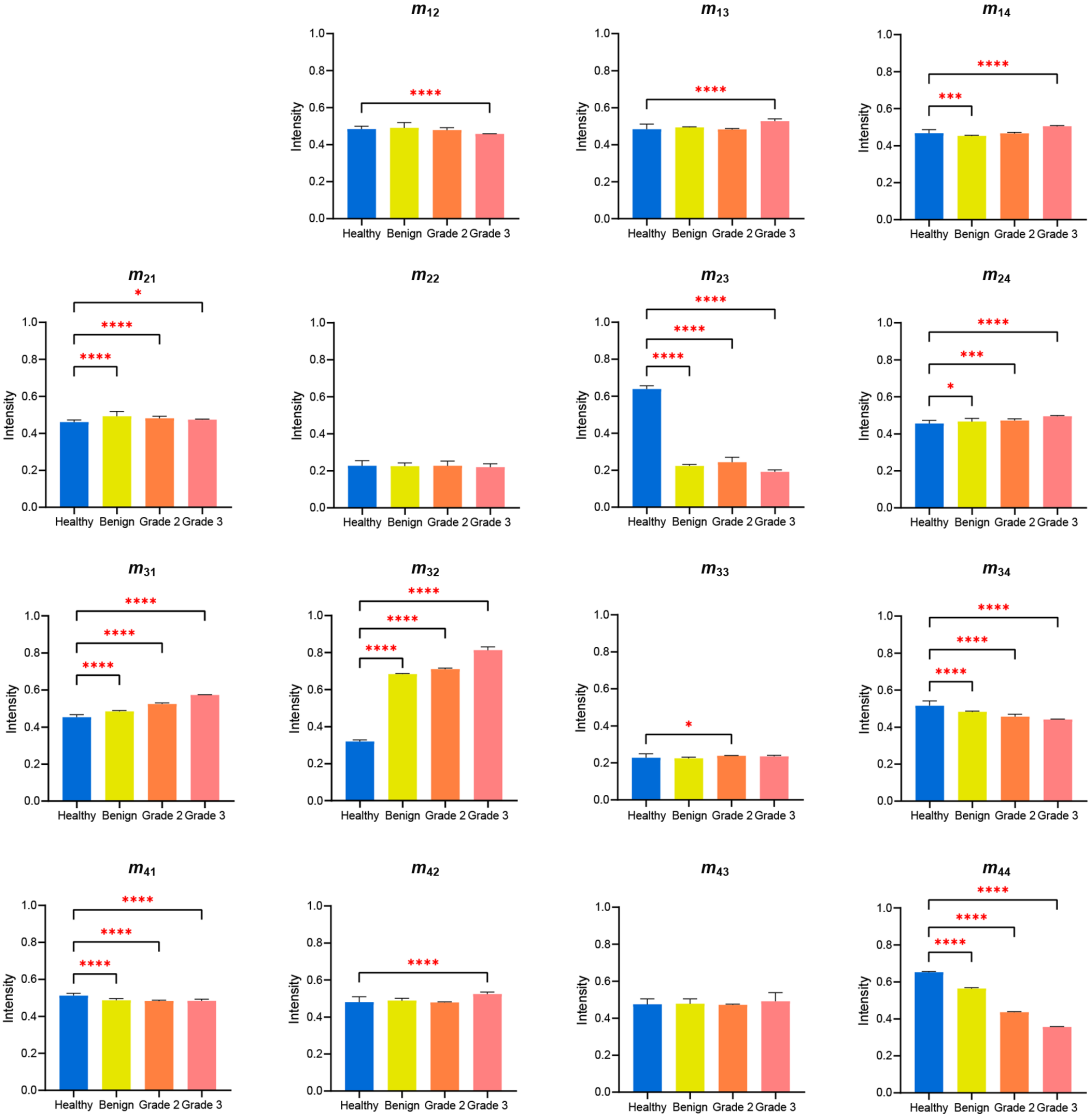
One-way ANOVA results for Mueller matrix element intensities of healthy and cancerous samples. The star symbols represent the *p*-values: 0.033 (*), <0.001 (***), <0.0001 (****), as determined by paired *t*-test.

**Fig. 15:**
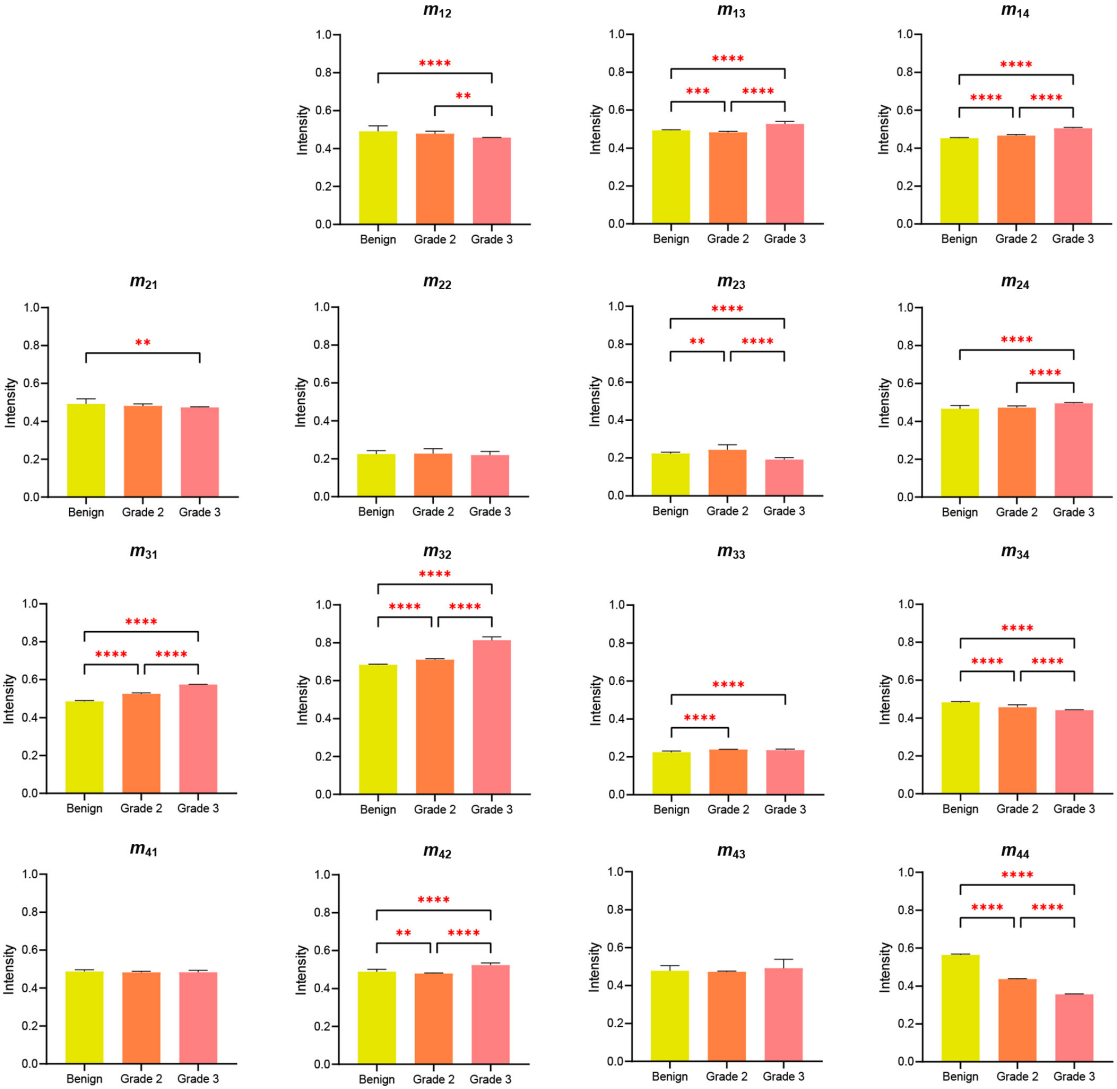
One-way ANOVA results for Mueller matrix element intensities of cancer samples. The star symbols represent the *p*-values: 0.002 (**), <0.001 (***), <0.0001 (****), as determined by paired *t*-test.

Figures 14 and 15 show the one-way ANOVA test results for the intensity differences between the four sample classes (healthy, benign, grade-2, and grade-3) and three cancer sample classes (benign, grade-2, and grade-3), respectively. Referring to Fig. 14, the element intensities of the healthy sample differ from those of the three cancerous samples with a significance of p<0.05 for all elements other than $m_{22}$ and $m_{43}$. Elements $m_{23},m_{31},m_{32},m_{34}$, $m_{41}$, and $m_{44}$ all show particularly significant differences in intensity between the healthy sample and the benign, grade-2, and grade-3 samples (p<0.0001). In particular, elements $m_{23},m_{32}$, and $m_{44}$ have significantly greater differences in intensities between the healthy sample and cancerous tissue group. Similarly, in Fig. 15, elements $m_{13},m_{14},m_{23},m_{31},m_{32},m_{34},m_{42}$, and $m_{44}$ show significant intensity differences between the three kind samples (benign, grade-2, and grade-3), with *p*-values ranging from <0.0001 to 0.002. It is quite surprising that there are a clear difference in intensity for each pair of elements *m*_31_, *m*_32_, and *m*_44_.

### 4.2 MMT Parameters

Table 3 shows the average MMT parameter values calculated from Eqs. (24) for the healthy, benign, grade-2, and grade-3 samples. The anisotropy indicator, A, has an explicit link to the anisotropy or isotropy of optical media.^34^ In particular, A reduces as the dispersion of the fiber orientation angle or order of alignment of the fibers decreases. In other words, a value of A closed to 1 indicates an presence of fibrous scatters and thus implies that the medium is anisotropic. The depolarization power factor, b, is a scatterer offset indicator, with a value determined by both the structure and the density of the scatterers.^34^ While b is associated with numerous media attributes, it is particularly sensitive to the scatterer size, particularly for scatterers with a dimension smaller than the wavelength of the incident light. Finally, parameter t is related to the magnitude of the anisotropy of the sample.^34^ In other words, t is sensitive to the structure of anisotropic media. In practice, t is closely related to b and varies in the interval of [0,b], where 0 and b correspond to perfectly isotropic and perfectly anisotropic materials, respectively.

**Table 3:** MMT parameters A,b,t, and depolarization powers for healthy and cancerous human breast tissue samples.

Sample	Anisotropy indicator (*A*)	Depolarization power factor (*b*)	Index related to magnitude of anisotropy (*t*)	Depolarization powers (Δ)
Healthy	0.7721	0.2278	0.6418	0.4459
Benign cancer	0.7942	0.2245	0.6750	0.4931
Malignant cancer (grade 2)	0.7866	0.2191	0.6784	0.549
Malignant cancer (grade 3)	0.7510	0.2121	0.7102	0.5944

The results presented in Table 3 show that all four sample classes have an anisotropy indicator value of A closed to 0.7. In other words, all of the samples, both healthy and cancerous, are anisotopic. Furthermore, parameter b reduces with an increasing cancer severity, whereas parameter t increases. Thus, it is inferred that the depolarization effect intensifies as the cancer severity increases.

For optical media, a lower value of the depolarization power (?) indicates a greater isotropy. Conversely, a higher depolarization power indicates a greater anisotropy.^36^ Table 3 presents the depolarization powers of the present samples, as calculated using Eq. (5). The healthy sample has a depolarization power of Δ=0.4459 and is thus confirmed to be more isotropic, with a simple microstructure. By contrast, the grade-3 malignant cancer sample has a depolarization power of Δ=0.5944, which indicates that the sample has a more anisotropic and complicated microstructure.

### 4.3 FDHs of Mueller Matrix Element Intensities

**Fig. 16:**
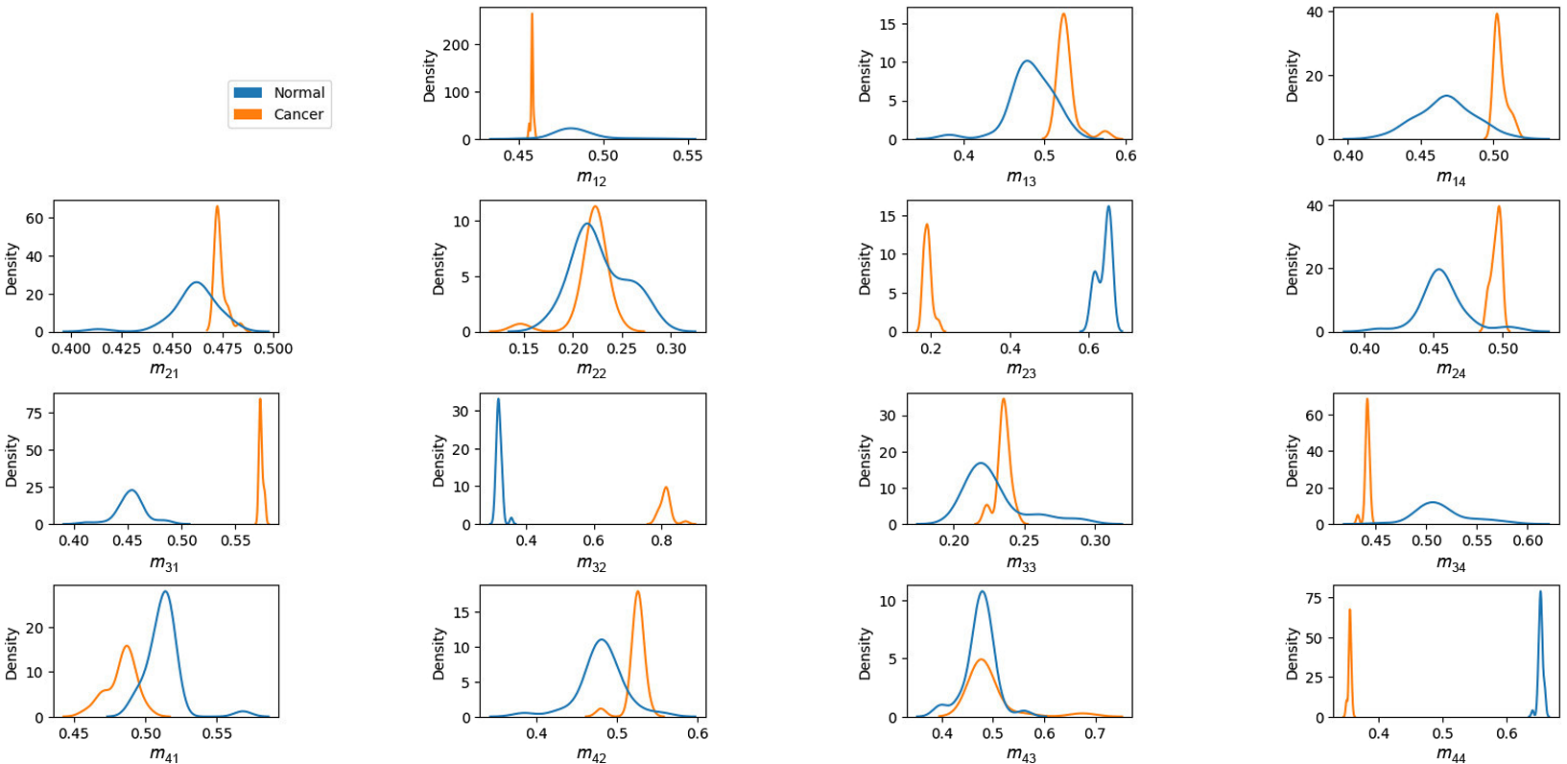
FDHs of pixel intensity of each Mueller matrix element for healthy and cancerous human breast tissue samples.

Figure 16 shows the FDHs of the 16 elements in the Mueller matrix images of the healthy and cancerous tissue samples. As expected, the FDHs of the two classes are widely separated for the $m_{23},m_{32}$, and $m_{44}$ elements, with the distributions of the healthy samples centered at higher intensity values for the $m_{23}$ and $m_{44}$ elements and a lower intensity value for the $m_{32}$ element. Interestingly, the results suggest that the intensity of element $m_{31}$ may also provide a feasible means of differentiating between healthy and cancerous samples.

**Fig. 17:**
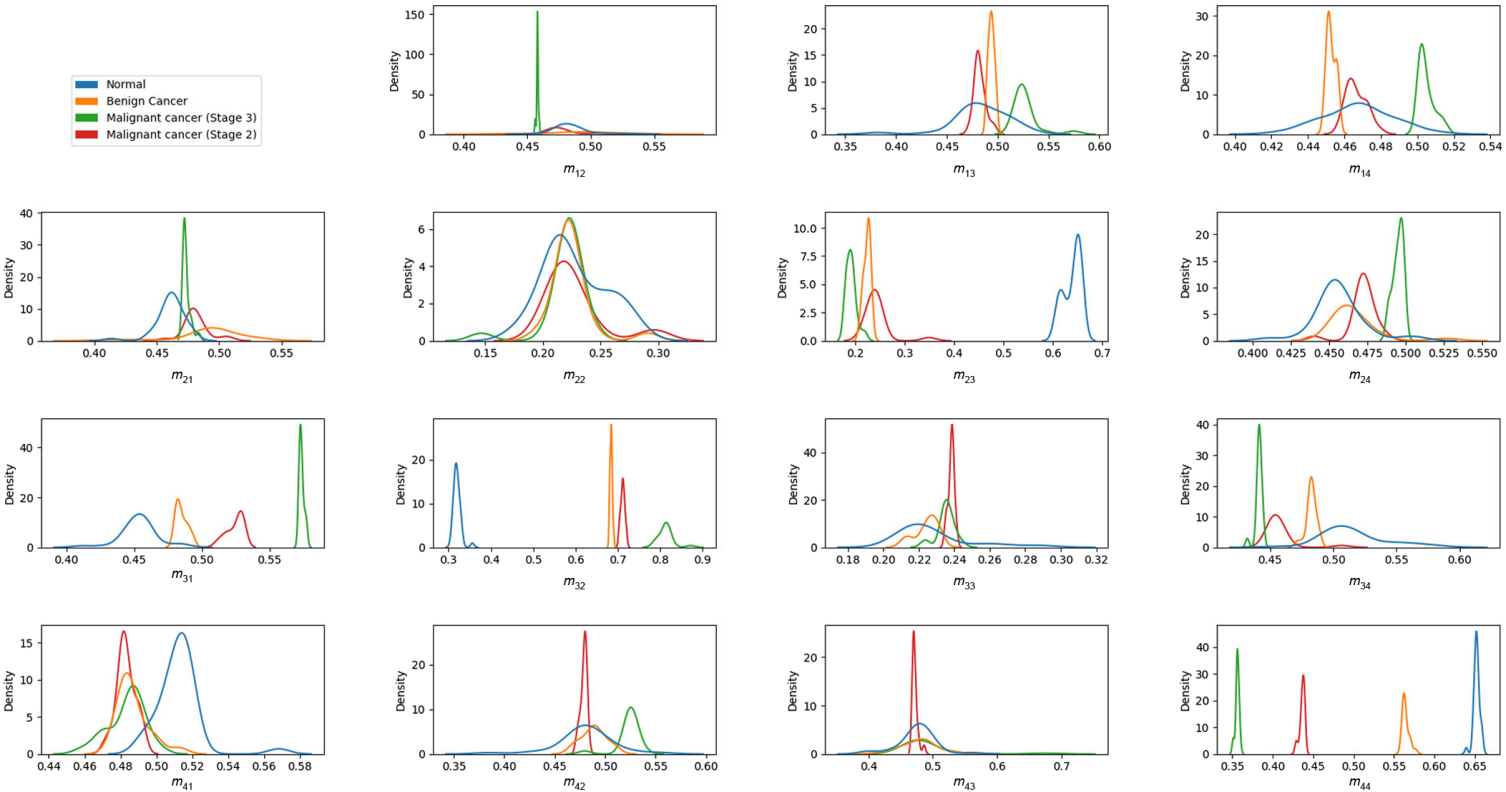
FDHs of pixel intensity of each Mueller matrix element for healthy, benign, grade-2 malignant, and grade-3 malignant samples.

Figure 17 compares the intensity FDHs of the healthy, benign, grade-2 malignant, and grade-3 malignant samples. It is seen that the FDHs of the four classes are well-spaced for the $m_{32}$ and *m*_44_ elements. Specifically, at position $m_{32}$, all four lines do not overlap, the blue line (healthy group) deviates completely to the left while the remaining three lines are arranged in a separate order and clearly deviate to the right. Similarly at $m_{44}$, a signal is authentic when all four lines are clearly separated and located in each separate region. In other words, the intensities of these two elements are particularly suitable for distinguishing between the four sample classes.

It can be seen that normal cells are usually uniform in size and shape, with a well-defined nucleus, whereas cancer cells can vary greatly in size and shape, and their nuclei may be enlarged or deformation. These cause different changes in polarization characteristics including birefringence, dichroism, and depolarization, leading the distinctions of polarization images among types of samples. For example, the structure of cartilage or tendons exhibits birefringence while collagen fibers exhibit arbitrary orientation.^37^^,^^38^ The depolarization also depends on the different morphological parameters of tissue such as the density, its distribution, size, shape, and refractive index of the tissue scatterers.^21^^,^^26^ The polarization capacities of biological tissues, nanoparticles, and other materials have all been studied using the Mueller matrix approach, which has been used to identify the characteristics of various sample types. As a result, the Mueller matrix method advances light science in medicine, especially in the areas of disease and cancer diagnosis. It could lead to the development of economical and noninvasive diagnostic methods in the near future.

Overall, the results presented above indicate the potential for using MMP to differentiate between healthy and cancerous human breast tissue samples. It is noted that performing polarization measurements on thin histology slides of breast cancer tissue is the first step of the proposed study in finding differences in polarization characterizations among types of breast tissue at different levels of cancers. Once this result has been confirmed and proven, the study will be carried out in an anesthetized mouse model without the need to remove the sample from the tumor. However, it should be noted that the present trials have considered only a relatively small sample size (n=20 to 35) for each sample class. A small sample size is associated with greater variability and lower dependability since it may contribute to bias.^39^ As a result, future studies should repeat the trials with a larger sample size to corroborate the present findings. In addition, the measurement process used in the present study is time-consuming (from 10 to 15 min) since the polarization states of the polarizers are adjusted one by one under motor-driven rotation stages. Thus, reducing the measurement time by a software solution for automatic control of the motor-driven rotation stages is necessary.

## 5 Conclusions

Optical diagnostic techniques have attracted significant attention in the literature as a means of evaluating a wide range of substances, including textiles and biological tissues, due to their nondestructive properties, speed, and affordability. The present study has used a backscattering MMP system to classify four classes of human breast tissue samples: healthy, benign, grade-2 malignant, and grade-3 malignant. The observation results have shown that the average intensities of the $m_{23},m_{32}$, and $m_{44}$ elements of the Mueller matrix image provide a reliable means of distinguishing not only between healthy and cancerous samples but also of estimating the grades of cancerous samples as benign, grade-2 malignant, or grade-3 malignant. The ANOVA test results have confirmed that the intensities of these three elements show significant differences (p<0.0001) among the four classes of samples. The FDHs of the element intensities have suggested that elements $m_{32}$ and $m_{44}$ are particularly effective indicators of the sample class. The tendencies of the MMT parameters derived for the four classes are consistent with the observation and statistical results. Overall, the results presented in this study suggest that backscattering MMP has significant potential as a tool for aiding pathologists in diagnostic applications for breast cancer.

## Data Availability

No materials were used for the analysis. The code and data used to generate the results are available in the Code Ocean repository: https://doi.org/10.24433/CO.7469965.v1.
